# Microbial insights into ruminal fiber degradation and feed efficiency of Hu sheep

**DOI:** 10.3389/fmicb.2025.1561336

**Published:** 2025-04-22

**Authors:** Haoyu Xu, Guoxiu Wang, Qihao Gao, Zhen Liu, Jiale Jia, Yunfei Xu, Zhanyu Chen, Baosheng Li, Chong Li

**Affiliations:** ^1^College of Animal Science and Technology, Gansu Agricultural University, Lanzhou, China; ^2^Gansu Runmu Bio-Engineering Co., Ltd, Yongchang, Gansu, China

**Keywords:** sheep, rumen fiber degradation, feed conversion efficiency, rumen microbiota, growth traits

## Abstract

Ruminal fiber degradation is essential for feed conversion efficiency in sheep; however, it remains unclear whether individual variations in ruminal fiber degradation directly affect feed conversion efficiency. Here, the relationship between ruminal fiber degradation rate and feed conversion efficiency and influence of rumen structure, function, and microbiota on fiber degradation were investigated. A total of 190 male Hu lambs were randomly selected, raised from birth to 180 days, and slaughtered. The relationships between ruminal fiber degradation rate and feed conversion efficiency, growth performance, and ruminal fermentation parameters were analyzed. Key microorganisms influencing ruminal fiber degradation were identified using multiple methods: microbial wide association study, correlation analysis, and differential abundance analysis. Both neutral detergent fiber (NDF) and acid detergent fiber (ADF) degradation rates were significantly correlated with feed conversion efficiency and intake. Seven genera were closely associated with NDF degradation rate: 6 belonged to Firmicutes (*Anaerotruncus*, *Family_XIII_UCG-002*, *Lachnoclostridium_1*, *Moryella*, *Ruminococcaceae_NK4A214_group*, and *Veillonellaceae_UCG-001*); 1, Bacteroidetes (*Prevotellaceae_UCG-003*). Eight genera were closely associated with ADF degradation rate: 6, Firmicutes (*Lachnospiraceae_ND3007_group*, *Family_XIII_UCG-002*, *Lachnoclostridium_1*, *Lachnospiraceae_UCG-002*, Moryella, and *Ruminococcaceae_NK4A214_group*); 1, Bacteroidetes (*Prevotellaceae_UCG-003*); and 1, Actinobacteria (*Olsenella*). In conclusion, high ruminal fiber degradation rates significantly enhance feed conversion efficiency, with specific microbial genera from the phylum Firmicutes and family Lachnospiraceae playing pivotal roles in fiber utilization. These findings provide a microbial basis for optimizing rumen fiber degradation efficiency in sheep and highlight the potential of uncultured taxa as future targets for improving feed conversion efficiency.

## 1 Introduction

The economic benefits of feedlot-fattened sheep depend significantly on growth performance and feed conversion efficiency. Feed cost is a critical factor that impacts profitability in the livestock industry, often accounting for more than half of the total production costs in large-scale feedlot systems for sheep (Li et al., 2024). To improve the production efficiency of sheep, producers need to enhance the conversion rate of feed into body weight (BW), known as feed efficiency (Nkrumah et al., 2006; Welch et al., 2021). Typically, feed conversion efficiency is indirectly measured by the feed-to-gain ratio (van Kempen et al., 2023), a key metric that impacts growth rate and other performance traits (Le Graverand et al., 2023). Identifying factors that influence feed conversion efficiency is essential for developing nutritional strategies that optimize this efficiency.

Primary factors that affect feed efficiency include feed quality and composition, rearing environment, and breed of sheep. However, even under identical conditions for feed, management, and breed, fattening sheep show considerable individual differences in feed efficiency. These differences may stem from various factors, such as variation in nutrient digestion and absorption in the rumen and intestines as well as nutrient transport and utilization. Cellulose and hemicellulose are essential degradable carbohydrates in ruminant diets that are broken down by rumen microorganisms into short-chain fatty acids (SCFAs), which are absorbable energy sources for the host (Singh et al., 2024). Rumen microbial communities play a crucial role in feed conversion by breaking down dietary fiber into metabolizable nutrients (Lovendahl et al., 2018). Previous studies have shown that the use of exogenous cellulase or hemicellulase can improve fiber degradation rates, thereby enhancing feed efficiency in ruminants (Vittorazzi et al., 2021; Guo et al., 2022). However, it is still unclear whether individual differences in ruminal fiber degradation directly influence feed efficiency in sheep.

The ability of ruminants to degrade plant fiber relies on their gastrointestinal microbiota. Individual sheep differ in their digestive efficiency of plant-based feed, primarily because of variations in rumen microbial communities (Huhtanen et al., 2015; Cabezas-Garcia et al., 2017). Recently, on the basis of in-depth research on the relationship between rumen microbiota and feed efficiency, scholars have proposed new strategies to select high-efficiency livestock according to their rumen microbial profiles (Jewell et al., 2015). This approach relies on identifying rumen microorganisms closely linked to the feed efficiency phenotype (Zhang et al., 2020). Fiber-degrading bacteria in the rumen microbiota produce cellulase and other enzymes that break down complex carbohydrates like cellulose and hemicellulose into SCFAs. In addition to primary fiber-degrading bacteria, some non-fiber-degrading bacteria can indirectly influence fiber degradation efficiency through co-metabolism and interactions. For instance, some non-fiber-degrading microorganisms can establish synergistic relationships with fiber-degrading bacteria through the exchange of fermentation products and utilization of metabolic by-products, thereby influencing the overall fiber-degradation capacity of the rumen. Certain hydrogen- and acid-producing bacteria can assist other fiber-degrading microorganisms in efficiently utilizing cellulose resources, thus promoting the degradation of cellulose in the diet (Nosalova et al., 2024). Furthermore, interactions between the host and microbiota significantly influence fiber degradation in the rumen. The host’s rumen environment, including physical structure, pH, and redox conditions, modulates microbial colonization and activity, thereby impacting fermentation and feed efficiency (Hristov et al., 2019). For example, changes in gastrointestinal pH can alter rumen microbial community structure and affect fiber degradation rates (Pucetti et al., 2024). In turn, microbial communities can influence the host’s metabolism, immunity, and feed efficiency through metabolic products and signaling molecules.

Although many fiber-degrading genera, such as *Ruminococcus* and *Fibrobacter*, have been studied extensively, the complexity of rumen microbial communities sur-passes our current knowledge. Numerous microbial populations that may play essential roles in fiber degradation remain under-researched or uncultured. Recently, high-throughput 16S rRNA sequencing has revealed novel microbial taxa that may be significantly associated with fiber degradation efficiency (Zhao et al., 2022); however, they have not yet been effectively isolated and characterized because of culturing limitations. Imbalances in microbial communities or absence of key species can significantly reduce fiber degradation, affecting nutrient absorption and feed efficiency. Given the complexity of the rumen microbiome, microbial populations that influence fiber degradation in sheep have not yet been elucidated.

Therefore, ruminal fiber degradation plays a crucial role in the feed conversion efficiency of sheep. However, factors that influence feed efficiency are multifaceted, and it is still unclear whether individual differences in ruminal fiber degradation rates directly impact feed efficiency. Our hypothesis was that specific ruminal microorganisms are key regulators of fiber degradation and, thereby, influence feed efficiency in sheep. The objectives of this study were to investigate the effects of ruminal fiber degradation rates on feed efficiency and growth performance of Hu sheep, characterize the rumen microbiota of sheep with varying fiber degradation rates, evaluate the association between ruminal microbial community features and individual differences in feed efficiency, and identify key microorganisms involved in fiber degradation. Our aim was to provide a scientific basis for optimizing feed conversion efficiency in sheep.

## 2 Materials and methods

### 2.1 Ethical statement

The animal procedures used in this study were reviewed and approved by the Academic Committee of Gansu Agricultural University, in accordance with the guidelines established by the Gansu Provincial Committee for the Care and Use of Biological Research Animals (Approval No. GSAU-Eth-AST-2021-021).

### 2.2 Experimental design

A total of 190 male Hu sheep, of similar age, good physical condition, and comparable weaning weights (15.91 ± 2.70 kg), were randomly selected for this study. The sheep were provided by Defu Agricultural Co. Ltd. (Minqin, China). The formal feeding trial started at 80 days of age and continued until 180 days of age, at which point all lambs werCe slaughtered. The rumen contents were collected, and parameters such as neutral detergent fiber (NDF), acid detergent fiber (ADF), NDF degradation rate (NDFD), and ADF degradation rate (ADFD) were measured. The correlations between these fiber degradation rates and various parameters, such as growth performance, feed intake, and feed conversion efficiency, were analyzed. The lambs were categorized into high and low NDFD and ADFD groups based on their respective degradation rates. The NDFD and ADFD values were separately ranked, and outliers exceeding the mean ± 2 standard deviations (SD) were excluded to ensure the rationality of the data distribution. Subsequently, the 10 individuals with the highest and lowest NDFD values were assigned to the high NDFD (H-NDFD) and low NDFD (L-NDFD) groups, respectively. Similarly, the 10 individuals with the highest and lowest ADFD values were assigned to the high ADFD (H-ADFD) and low ADFD (L-ADFD) groups, respectively. Among the selected lambs, seven exhibited both H-NDFD and H-ADFD, while six exhibited both L-NDFD and L-ADFD. Differences in various parameters between the high and low degradation rate groups were compared to validate the results of the correlation analysis. Rumen bacterial genera abundance was analyzed in all lambs using 16S rDNA sequencing. To ensure the reliability of the results, three methods were used to identify key genera associated with rumen fiber degradation rates: correlation analysis, microbiome-wide association study (MWAS), and differential abundance analysis between the high and low degradation rate groups. The overlapping genera identified by all three methods were considered as key microbiota linked to fiber degradation.

### 2.3 Animal management and sample collection

All lambs were reared with their mothers prior to weaning and were supplemented with starter feed at 7 days of age; they received a standardized immunization protocol. Lambs had ad libitum access to starter feed and water. Weaning occurred at 56 days of age, after which all lambs were housed individually in pens (each pen, 0.8 m^2^) to facilitate specific measurements. All lambs were managed under identical conditions. A 14-day transition period followed weaning, during which the diet was gradually shifted from starter feed to a total mixed ration. The starter feed and total mixed pellet fattening feed were produced by Gansu Runmu Biological Engineering Co., Ltd. (Jinchang, Gansu, China), and the formulation and nutritional composition are detailed in Supplementary Table S1. After an initial 10-day trial period, the experiment officially began at 80 days of age. Body weight (BW) was measured every 20 days, using a calibrated electronic scale, with measurements taken in the morning before feeding to ensure consistency. Average daily gain (ADG) was calculated for each 20-day interval using the formula: ADG = (BW at the end of the interval − BW at the start of the interval) / number of days in the interval. Feed intake (FI) was measured by recording the amount of feed provided and the residual feed. The feed conversion rate (FCR) was calculated using the following formula: FCR = FI/ADG.

At 180 days, all experimental lambs were slaughtered over a 3-day period. After a 12 h fasting period, the lambs were weighed and subsequently transported to the experimental slaughter house, where they were euthanized by severing the jugular vein and carotid artery. Next, the rumen contents were collected and mixed thoroughly, and the pH was immediately measured using a pH meter (Sartorius PB-10; Sartorius Biotech Inc., Göttingen, Germany). The mixed rumen contents were stored in sterile tubes at −80°C for analyses of rumen microbiota and fiber degradation rates. The contents were filtered through four layers of sterile medical gauze, packaged in cryovials, and stored at −20°C for analyses of rumen fermentation parameters. Then, the rumen was weighed, and tissue samples were obtained from the location of the cranial ventral sac and fixed in 4% paraformaldehyde for morphological measurements.

### 2.4 Measurement of rumen fiber degradation rates

We assessed the degradation rates of NDF and ADF in the rumen after a 12-h fasting period by using the acid-insoluble ash method (Huang et al., 2022). To perform this analysis, rumen contents and feed were dried at 65°C for 8 h, and NDF, ADF, and acid-insoluble ash contents were measured. NDF and ADF contents were analyzed using the protocol of Van Soest et al. (1991), whereas acid-insoluble ash content was determined using Soltani’s method (Soltani et al., 2020). The degradation rates of NDF and ADF were calculated using the indirect digestibility approach, with acid-insoluble ash as the internal marker. The formula for determining NDF and ADF degradation rates was as follows:


$$NDF/ADF\;\mathrm{degradation rate}\;\left(\%\right)=\left[1-\left(A/B\right)\times \left(FB/FA\right)\right]\times 100\text{,}$$

where A represents acid-insoluble ash concentration in the feed, B denotes its concentration in the rumen contents, FA is NDF/ADF concentration in the feed, and FB is NDF/ADF concentration in the rumen contents.

### 2.5 Determination of rumen fermentation parameters

Volatile fatty acids (VFAs) in the rumen fluid were quantified using gas chromatography. Initially, the rumen fluid samples were pretreated by filtering through a 0.45 μm disposable filter. The clarified supernatant was transferred to vials for gas chromatography analysis. The VFA concentrations were quantified using a Shimadzu gas chromatograph (GC-2010Plus, Japan), with 2-ethylbutyric acid used as the internal standard. The carrier gas is high-purity nitrogen, with an injection port temperature of 230°C. The split ratio is 40:1, and the injection volume is 0.1 mL. The column flow rate is 3.0 mL/min. The initial temperature of the column oven is 60°C, then it is ramped up to 120°C at a rate of 10°C/min and held for 2 min, followed by ramping up to 180°C at a rate of 15°C/min and held for 5 min (Cottyn and Boucque, 2002).

### 2.6 Histomorphology of the rumen

**Figure 1:**
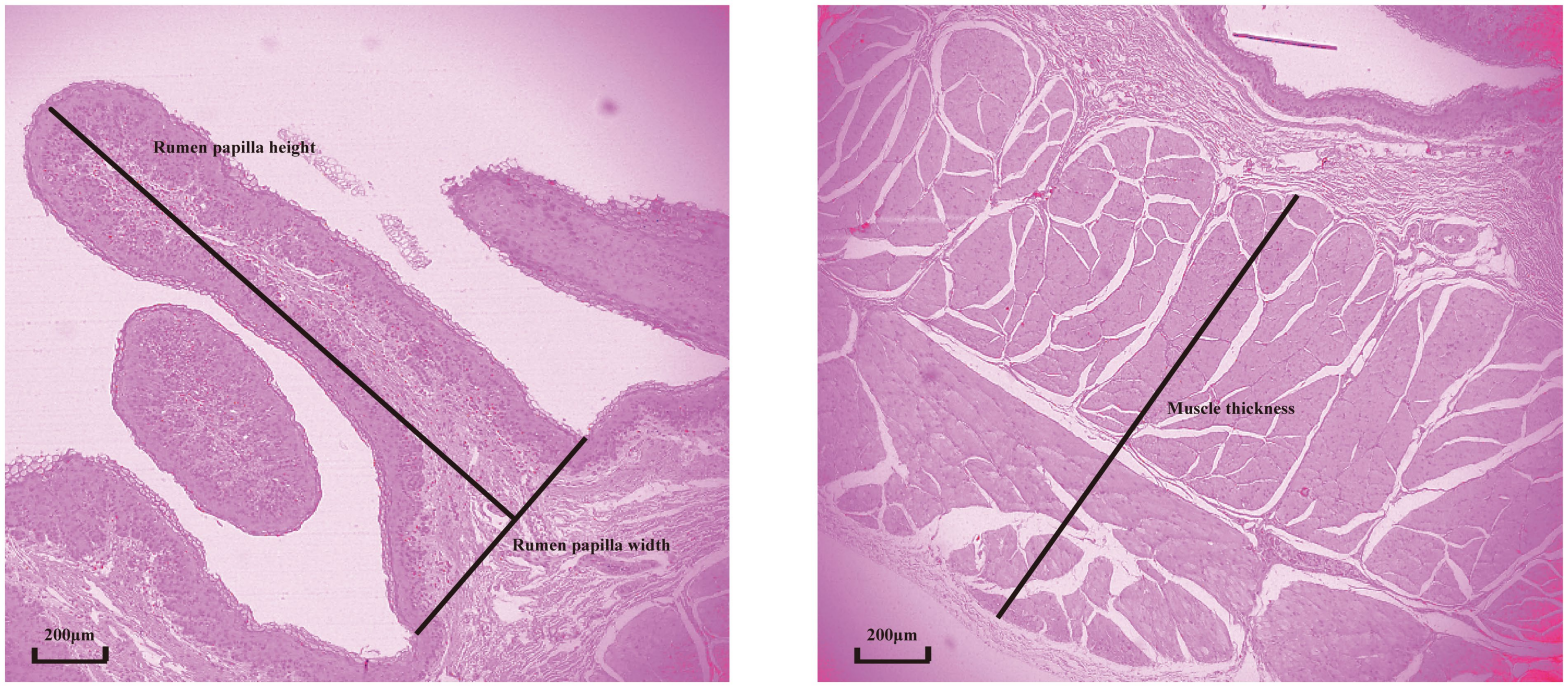
A schematic diagram for the determination of rumen tissue morphology.

The rumen samples, fixed in 4% paraformaldehyde, were dehydrated in a graded ethanol series and embedded in paraffin. The embedded tissues were sectioned at 5 μm thickness using a microtome and stained with hematoxylin–eosin (Huang et al., 2022). For each ruminal cross-section, five intact well-oriented papillae were selected in triplicate for each ruminal cross-section. Papilla length and width and muscle layer thickness were determined using an image analysis system (Motic Image Plus 2.0; Motic China Group Co. Ltd., Xiamen, China). Papilla length was measured from the apex to the base of the papilla along its axis, and papilla width was measured at the bottom of the papilla. Muscle layer thickness was measured from the junction between the submucosal and muscular layers to that between the muscular layer and tunica serosa. A schematic diagram illustrating the histological sections and the determination of rumen tissue morphology is provided in Figure 1.

### 2.7 16S rDNA sequencing

Total DNA was extracted from rumen content samples for 16S rDNA sequencing by using the Magnetic Stool DNA Kit (Tiangen Biotech, Beijing, China; Catalog No.: DP 712). DNA concentration and purity were checked using 1% agarose gel electrophoresis. The DNA was diluted to 1 ng/μL by using sterile water. PCR amplification was performed using primers targeting the V3-V4 region of the 16S rRNA gene (515-F: CCTAYGGGRBGCASCAG and 806-R: GGACTACNNGGGTATCTAAT) (Chong et al., 2022). The PCR volume contained 15 μL of Phusion® High-Fidelity PCR Master Mix (New England Biolabs, Ipswich, MA, USA), 2 μM of forward and reverse primers, and approximately 10 ng of template DNA. The thermal cycling conditions were as follows: initial denaturation at 98°C for 1 min, followed by 30 cycles of denaturation at 98°C for 10 s, annealing at 50°C for 30 s, and extension at 72°C for 30 s and a final hold at 72°C for 5 min. The PCR products were mixed with SYBR Green dye and analyzed using 2% agarose gel electrophoresis. The PCR products were mixed in equal density ratios and purified using the Qiagen Gel Extraction Kit (Qiagen, Hilden, Germany). Sequencing libraries were prepared using the TruSeq® DNA PCR-Free Sample Preparation Kit (Illumina, San Diego, CA, USA), according to the manufacturer’s instructions, and indexed. Library quality was assessed using a Qubit 2.0 fluorometer (Thermo Scientific, Waltham, Massachusetts, USA) and an Agilent Bioanalyzer 2,100 system (Agilent, Santa Clara, CA, USA). Finally, the libraries were sequenced on the Illumina NovaSeq platform to generate paired-end reads of 250 bp.

### 2.8 Bioinformatics analysis

The paired-end reads were assigned to samples on the basis of their unique bar-codes and then truncated by removing the barcode and primer sequences. The FLASH software (Version 1.2.11^1^) (Tanja and Salzberg, 2011) as utilized to merge the paired-end reads when at least some overlaps occurred between reads generated from the corresponding ends of the same DNA fragment. The merged sequences were referred to as raw tags. Quality filtering of the raw tags was performed using fastp (Version 0.23.1) to obtain high-quality clean tags (Bokulich et al., 2013). The UCHIME algorithm was used to compare the clean tags against a reference database and detect and re-move chimeric sequences (Edgar et al., 2011), resulting in valid tags. The valid tags were denoised using the QIIME 2 software (Version QIIME 2–202,006) to obtain the initial amplicon sequence variants (ASVs) (default: DADA2), followed by filtering out ASVs with an abundance of less than 5 (Wang et al., 2021). The absolute abundance of ASVs was normalized using the standard sequence number corresponding to the sample with the fewest sequences, allowing for comparison in the composition differential analysis.

### 2.9 Statistical analysis

To assess correlations, Spearman’s rank correlation coefficient was calculated using the psych library in R software (version 4.3.1) to analyze the relationships between NDF and ADF degradation rates, growth traits, feed efficiency, ruminal fermentation parameters, and bacterial genera in ruminal contents. A correlation was considered moderate when *p* < 0.01 and ∣r∣ > 0.40, and weak when *p* < 0.01 and 0.20 < ∣r∣ < 0.40. A t-test was conducted in SPSS (version 27.0) between high and low fiber degradation rate groups to evaluate differences in growth traits, feed efficiency, ruminal fermentation parameters, and rumen histomorphometry. The t-test was performed using the stats library in R software (version 4.3.1) to determine significant differences at each classification level, with a statistical significance threshold of *p* < 0.05.

Microbial wide association study (MWAS) was utilized to identify bacterial taxonomic groups associated with ruminal fiber degradation in Hu sheep. The 16S rRNA gene sequencing data were subjected to quality control and preprocessing, including the removal of low-quality sequences, merging of overlapping sequences, and exclusion of chimeras, to obtain high-quality sequence data representative of the ruminal microbial community composition (such as ASVs). For marker genera, a filtered dataset at the genus level was used, and association analyses between 292 genera and ruminal fiber degradation rates were statistically assessed using a two-part microbial community range association model implemented with the dplyr and lmPerm libraries in R (version 4.3.1), as demonstrated by Wen et al. (2021) and Fu et al. (2015). The first part of the model addresses binary traits on the basis of the presence or absence of bacterial genera. Specifically, relative abundance greater than zero is coded as 1 (present) and equal to zero as 0 (absent), achieving a sample prevalence rate of less than 60%. The second part of the model focuses on quantitative traits and is typically used for regression analyses between the phenotype and abundance of bacterial genera, with a sample prevalence rate of 60% or more. The MWAS model is de-scribed as follows:


y=β1b+e

y=β2q+e

where y represents the ruminal fiber degradation rate. The first three principal components (PCs) are used as covariates in the association analyses to adjust for population substructure. β1 and β2 denote the regression coefficients for the binary and quantitative models, respectively; b stands for binary traits; q represents the centered log-ratio transformed abundance of bacterial genera; and e is the residual effect. The *p*-values were adjusted for multiple comparisons by using the Bonferroni correction, with the threshold *p*-value set at 0.01. The processed microbial community data were associated with ruminal fiber degradation data to evaluate the strength of the association between each microbial taxon and fiber degradation rate. On the basis of the adjusted significance level, microbial taxonomic groups significantly associated with ruminal fiber degradation were selected. The MWAS results were compared with the results of the Spearman correlation analysis to determine the overlapping taxonomic groups identified by the two methods.

## 3 Results

### 3.1 Correlation between fiber degradation rates in the rumen and growth traits and feed conversion efficiency of fattening lambs

**Figure 2:**
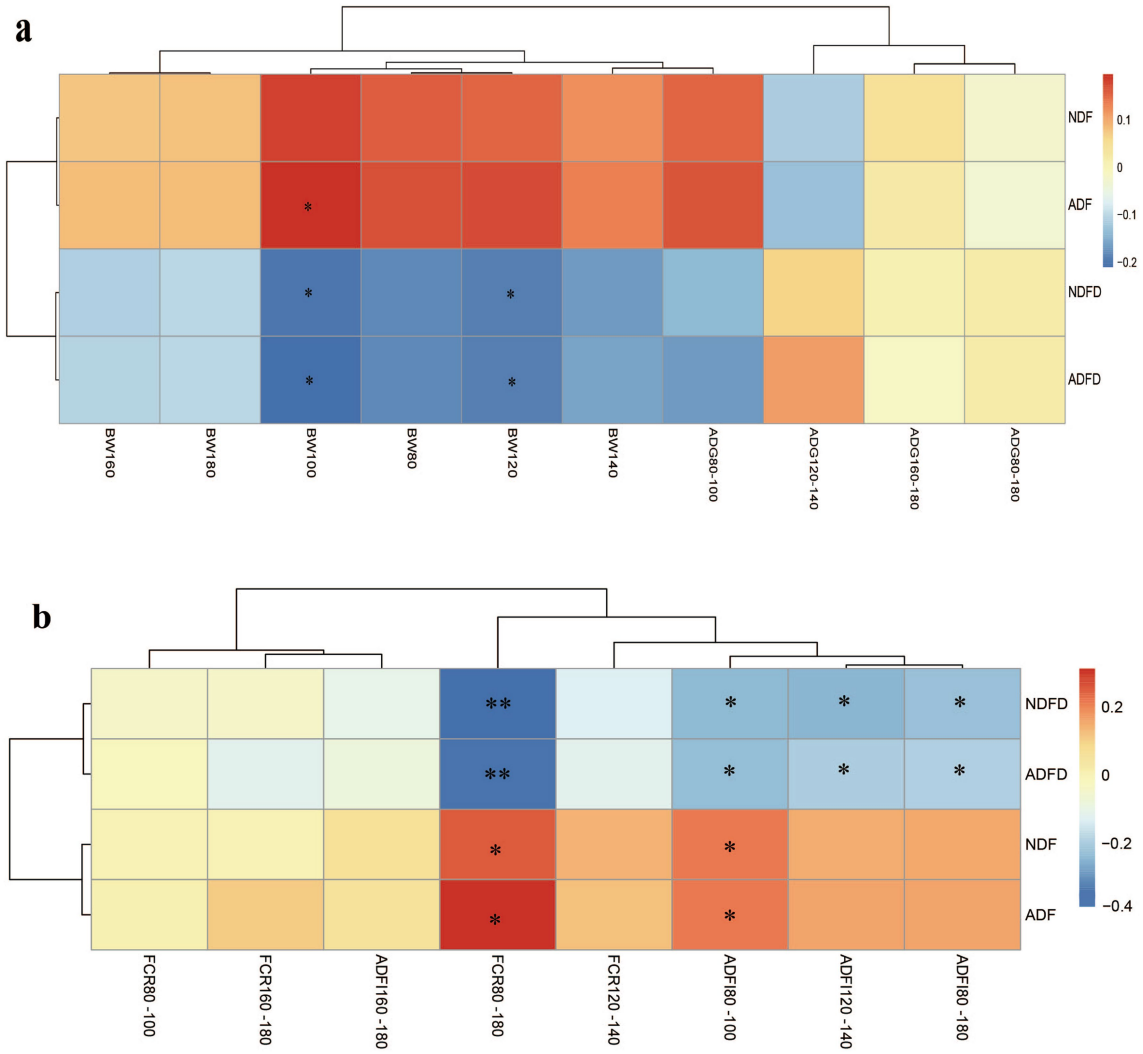
Correlation between fiber degradation rates in the rumen and growth traits and feed efficiency of fattening lambs. **(a)** Correlation between cellulose degradation rates in the rumen and growth traits of fattening lambs; **(b)** Correlation between fiber degradation rates in the rumen and feed efficiency of fattening lambs. The gradients in red and blue indicate positive and negative correlations, respectively. NDF, rumen neutral detergent fiber content; ADF, rumen acid detergent fiber content; NDFD, degradation rate of NDF; and ADFD, degradation rate of ADF. ** Denotes moderate correlation (*p* < 0.01, ∣*r*∣ > 0.40), whereas * denotes weak correlation (*p* < 0.01, ∣ r∣ >0.20).

To present the results clearly and avoid excessive data redundancy, representative data from the early (80–100 days), middle (120–140 days), and late (160–180 days) fattening stages were selected for inclusion in the manuscript. No moderate correlations were observed between any measured parameters and BW or ADG (Figure 2a). Weak correlations were detected for certain parameters, with ADF in rumen contents showing a significant positive correlation with BW at 100 days of age (*p* < 0.01, 0.20 < ∣*r*∣ < 0.40). Both NDFD and ADFD exhibited significant negative correlations with BW at 100 and 120 days of age (*p* < 0.01, 0.20 < ∣*r*∣ < 0.40).

Both NDFD and ADFD showed significant moderate negative correlations with FCR during 80–180 days (*p* < 0.01, ∣*r*∣ > 0.40; Figure 2b). No other moderate negative correlations were observed with FCR or ADFI. Weak negative correlations were found between NDFD and ADFD and ADFI during 80–100 days, 120–140 days, and 80–180 days. Additionally, NDF and ADF in rumen contents exhibited weak negative correlations with ADFI and FCR from 80 to 100 days (*p* < 0.01, 0.20 < ∣r∣ < 0.40).

To validate the correlation analysis results, lambs with the highest and lowest degradation rates of NDF and ADF were selected. Growth traits and feed efficiencies of the high NDF degradation rate (H-NDFD) group (*n* = 10) and low NDF degradation rate (L-NDFD) group (*n* = 10), as well as the high ADF degradation rate (H-ADFD) group (*n* = 10) and low ADF degradation rate group (L-ADFD) group (*n* = 10), were compared. The results indicate that BWs of the L-NDFD group at 80 and 100 days of age were significantly higher than those of the H-NDFD group (*p* < 0.05; Table 1). At 120 to 140 days of age, ADFI of the L-ADFD group was significantly greater than that of the H-ADFD group (*p* < 0.05), and FCR was also significantly higher in the L-ADFD group than in the H-ADFD group (*p* < 0.05). Throughout the period of 80 to 180 days, FCR of the L-NDFD group was significantly higher than that of the H-NDFD group (*p* < 0.05), and FCR of the L-ADFD group was also significantly higher than that of the H-ADFD group (*p* < 0.05). The differences in FCR from 80 to 180 days among the groups were consistent with the findings of the correlation analysis.

**Table 1:** Analysis of differences in growth performance and feed conversion efficiency of fattening lambs with different fiber degradation rates.

Item	Group		SEM	*p*-value	Group		SEM	*p*-value
	H-NDFD	L-NDFD			H-ADFD	L-ADFD		
BW, kg								
80 d	19.64	23.02	0.56	0.007	20.37	22.02	0.68	0.240
100 d	24.48	28.48	0.70	0.010	25.18	27.88	0.78	0.099
120 d	29.96	33.26	0.89	0.080	30.00	33.60	0.94	0.070
140 d	35.16	38.48	0.91	0.086	35.55	38.80	0.94	0.101
160 d	40.89	43.97	1.10	0.181	41.35	44.15	1.16	0.242
180 d	43.97	48.41	1.20	0.254	46.04	48.50	1.21	0.323
ADG, kg/d								
80–100 d	0.24	0.27	0.02	0.476	0.24	0.29	0.02	0.171
120–140 d	0.26	0.26	0.01	0.980	0.28	0.26	0.01	0.370
160–180 d	0.23	0.22	0.01	0.672	0.23	0.22	0.01	0.552
80–180 d	0.26	0.25	0.01	0.783	0.26	0.26	0.01	0.687
ADFI, kg/d								
80–100 d	1.06	1.29	0.05	0.057	1.10	1.31	0.06	0.095
120–140 d	1.50	1.71	0.05	0.070	1.51	1.78	0.05	0.017
160–180 d	1.75	1.91	0.07	0.244	1.76	1.91	0.06	0.254
80–180 d	1.47	1.67	0.05	0.082	1.48	1.71	0.06	0.053
FCR								
80–100 d	4.72	5.30	0.37	0.445	4.85	4.74	0.28	0.841
120–140 d	5.91	6.70	0.31	0.215	5.60	7.00	0.30	0.032
160–180 d	7.52	9.57	0.58	0.093	7.55	9.99	0.66	0.079
80–180 d	5.67	6.64	0.11	<0.001	5.77	6.51	0.13	0.009

H-NDFD, high NDF degradation rate group; L-NDFD, low NDF degradation rate group; H-ADFD, high ADF degradation rate group; L-NDFD, low ADF degradation rate group; BW, body weight; ADG, average daily gain; ADFI, average daily feed intake; FCR, feed conversion ratio.

### 3.2 Correlation between fiber degradation rates and fermentation parameters in the rumen of fattening lambs

**Figure 3:**
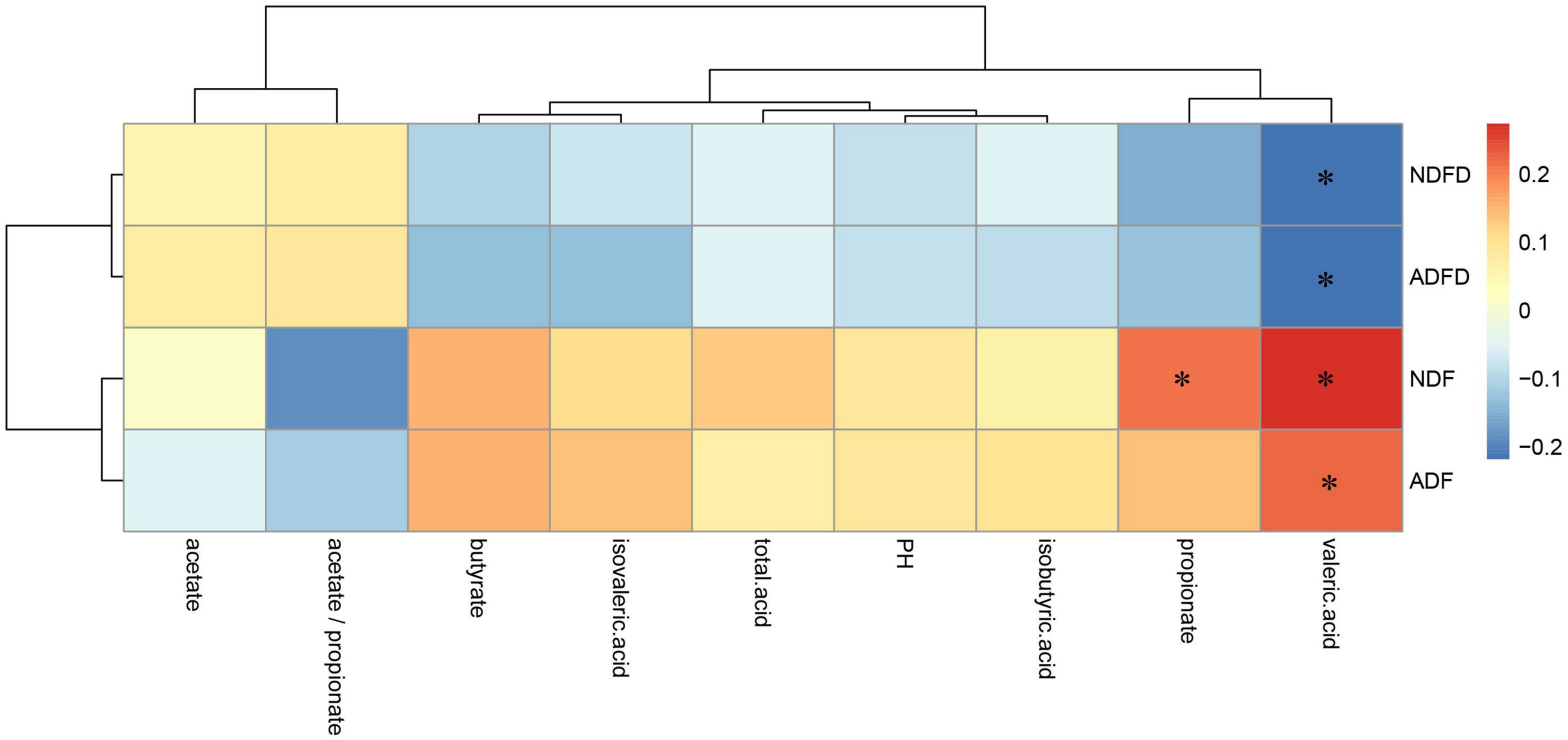
Correlation analysis of fiber degradation rates and fermentation parameters in the rumen of fattening lambs. NDF, rumen neutral detergent fiber content; ADF, rumen acid detergent fiber content; NDFD, degradation rate of NDF; and ADFD, degradation rate of ADF. ** Indicates moderate correlation (*p* < 0.01, ∣*r*∣ >0.40), whereas * denotes weak correlation (*p* < 0.01, ∣*r*∣ >0.20).

No moderate correlations were observed between fiber degradation rates and fermentation parameters (Figure 3). The NDF content exhibited weak positive correlations with the concentrations of propionic acid, and valeric acid (*p* < 0.01, 0.20 < ∣*r*∣ < 0.40). The ADF content was significantly positively correlated with the concentrations of valeric acid (*p* < 0.01, 0.20 < ∣r∣ < 0.40). Moreover, NDFD and ADFD showed a weak negative correlation with the concentrations of valeric acid (*p* < 0.01, ∣*r*∣ > 0.20).

To validate the correlation analysis results, we compared the differences in rumen fermentation parameters between the fiber degradation rate groups (Table 2). The results indicated that only the acetate/propionate ratio in the H-ADFD group was significantly higher than that in the L-ADFD group (*p* < 0.05). No significant differences were observed in the other fermentation parameters (*p* > 0.05).

**Table 2:** Analysis of differences in rumen fermentation parameters of fattening sheep with different fiber degradation rates.

Item	Group		SEM	*p*-value	Group		SEM	*P*-value
	H-NDFD	L-NDFD			H-ADFD	L-ADFD		
pH	5.82	6.05	0.11	0.309	5.69	5.90	0.10	0.293
Acetate (mmol/L)	14.82	17.14	2.70	0.673	16.68	17.67	2.79	0.862
Propionate (mmol/L)	7.72	13.44	2.07	0.185	7.68	12.79	2.01	0.220
isobutyric acid (mmol/L)	0.64	1.02	0.14	0.191	0.64	1.07	0.15	0.162
Butyrate (mmol/L)	5.39	6.98	0.81	0.340	5.45	7.10	0.88	0.363
isovaleric acid (mmol/L)	1.76	2.01	0.36	0.743	1.70	2.25	0.34	0.427
valeric acid (mmol/L)	0.83	1.58	0.19	0.071	0.87	1.40	0.19	0.182
total acid (mmol/L)	31.17	42.15	5.60	0.340	33.04	42.28	5.71	0.429
acetate/propionate	1.97	1.50	0.15	0.151	2.17	1.44	0.14	0.020

H-NDFD, high NDF degradation rate group; L-NDFD, low NDF degradation rate group; H-ADFD, high ADF degradation rate group; L-NDFD, low ADF degradation rate group.

### 3.3 Relationship between fiber degradation rates and rumen morphology of fattening sheep

We compared the differences in rumen morphology parameters between the fiber degradation rate groups (Table 3) and found no significant differences in any of the rumen morphology indices between the H-NDFD and L-NDFD groups as well as the H-ADFD and L-ADFD groups (*p* > 0.05).

**Table 3:** Analysis of differences in rumen morphology of fattening sheep with different fiber degradation rates.

Item	Group		SEM	*p*-value	Group		SEM	*p*-value
	H-NDFD	L-NDFD			H-ADFD	L-ADFD		
Rumen papilla height (μm)	1652.77	1790.33	176.85	0.702	1859.90	1742.24	168.76	0.731
Rumen papilla width (μm)	355.68	317.45	12.72	0.150	355.70	333.05	168.76	0.543
Muscle thickness (μm)	1430.02	1439.60	83.90	0.955	1503.88	1377.60	96.28	0.520

H-NDFD, high NDF degradation rate group; L-NDFD, low NDF degradation rate group; H-ADFD, high ADF degradation rate group; L-NDFD, low ADF degradation rate group.

### 3.4 Correlation between fiber degradation rates and rumen microbiota in fattening sheep

**Figure 4:**
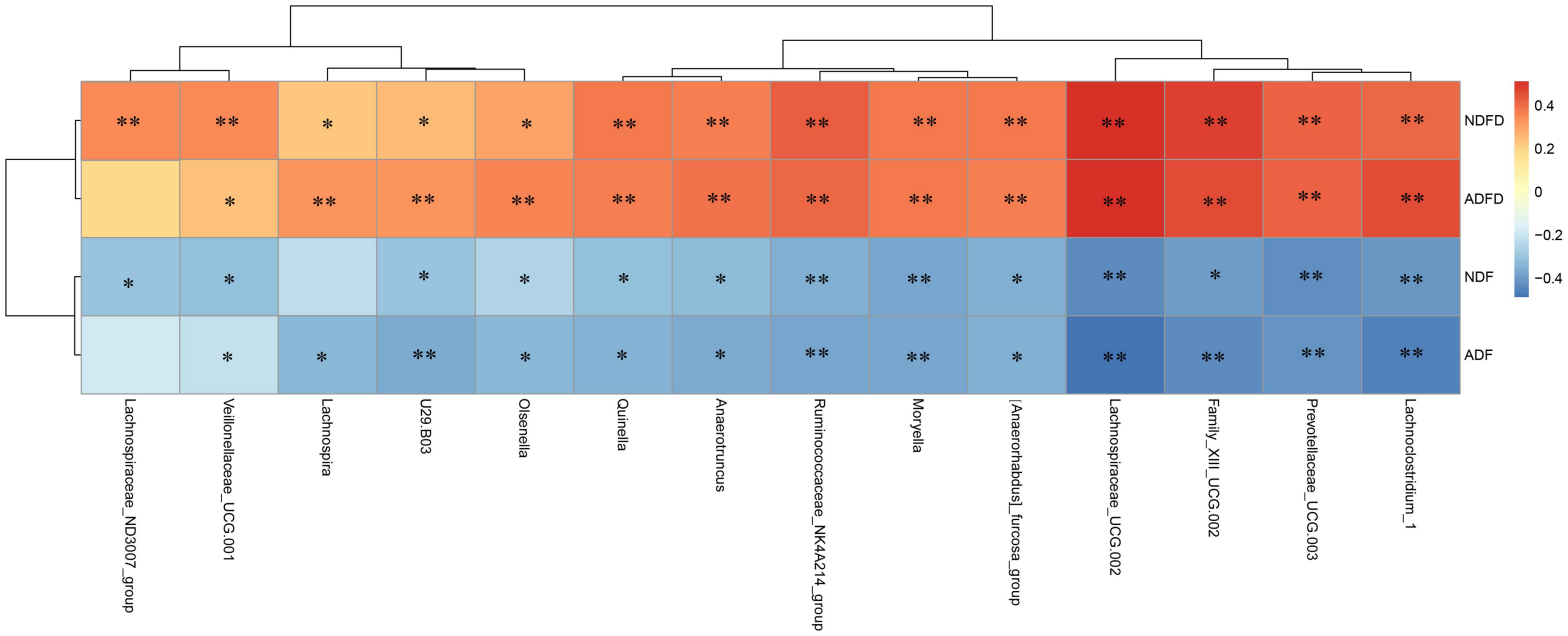
Correlation analysis of fiber degradation rates and rumen microbiota in fattening sheep. NDF, rumen neutral detergent fiber content; ADF, rumen acid detergent fiber content; NDFD, degradation rate of NDF; and ADFD, degradation rate of ADF. ** Indicates moderate correlation (*p* < 0.01, ∣*r*∣ >0.40), whereas * denotes weak correlation (*p* < 0.01, ∣*r*∣ >0.20).

We initially conducted a correlation analysis of the degradation rates of NDF and ADF, as well as their contents, with the relative abundance of bacterial taxa in the rumen contents of Hu sheep. Using a significance level of *p* < 0.01 and ∣*r*∣ > 0.40, we identified 14 taxa that were highly significantly correlated with either fiber degradation rates or fiber content (Figure 4).

**Figure 5:**
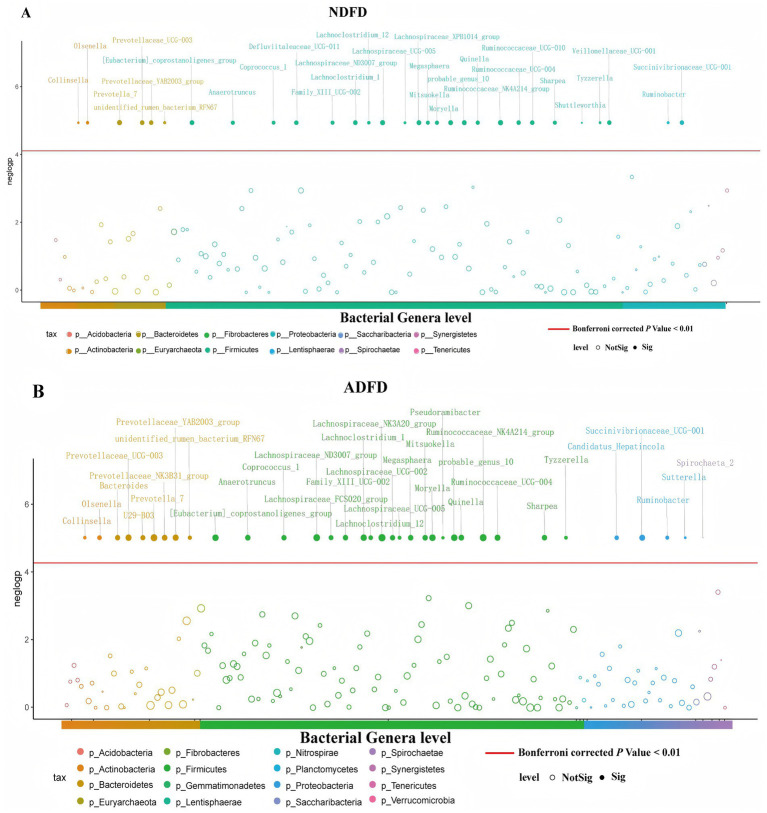
Identification of bacterial taxa associated with fiber degradation rates in the rumen via both MWAS and Spearman correlation analyses. **(A)** Bacterial taxa associated with NDFD; **(B)** Bacterial taxa associated with ADFD. NDFD, degradation rate of NDF; and ADFD, degradation rate of ADF.

We used MWAS to identify bacterial taxa significantly associated with fiber degradation rates in the rumen of Hu sheep. We compared these results with those from the Spearman correlation analysis, identifying the overlap between the taxa identified by both methods. We identified a total of 30 bacterial taxa related to NDFD (Bonferroni corrected *p* < 0.01; Figure 5A), and 22 taxa belonged to the phylum Firmicutes; 4, phylum Bacteroidetes; 2, phylum Actinobacteria; and 2, phylum Proteobacteria. Thirty-five bacterial taxa were significantly associated with ADFD (Bonferroni corrected *p* < 0.01; Figure 5B), with 21 belonging to Firmicutes; 7, Bacteroidetes; 4, Proteobacteria; 2, Actinobacteria; and 1, Spirochaetes. Of the 30 bacterial taxa significantly associated with NDFD via MWAS, 20 were found to overlap with those identified via Spearman correlation analysis. Within this overlap, 16 belonged to Firmicutes; 2, Bacteroidetes; and 2, Actinobacteria. Similarly, of the 35 bacterial taxa significantly associated with ADFD via MWAS, 23 were found to overlap via Spearman correlation analysis, including 16 from Firmicutes; 4, Bacteroidetes; 2, Actinobacteria; and 1, Proteobacteria. These overlapping taxa represent important microorganisms that consistently showed significant associations with fiber degradation rates across different analytical approaches.

**Figure 6:**
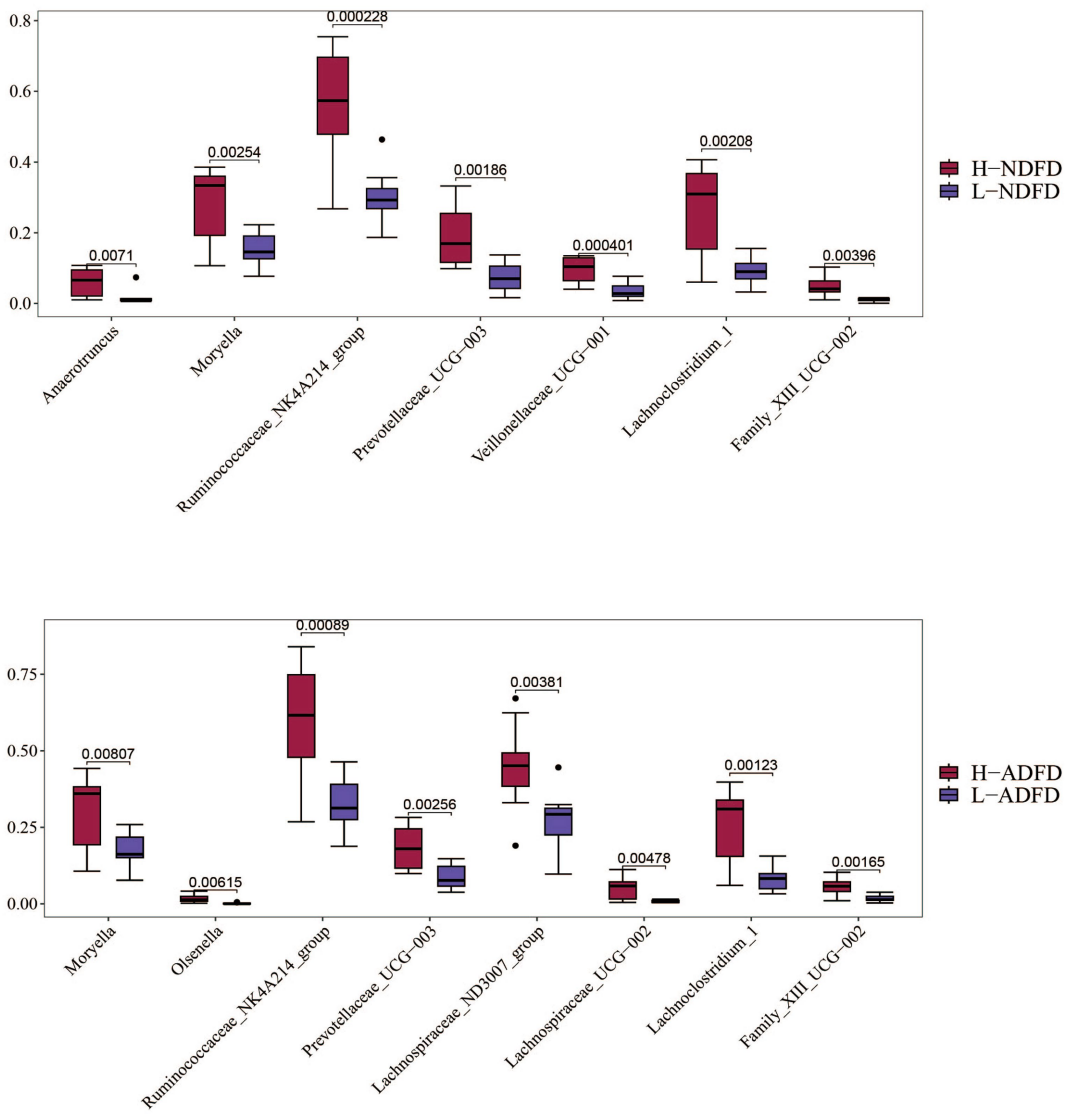
Analysis of differences in the relative abundance of microbial taxa between high and low detergent fiber degradation groups. NDFD, degradation rate of NDF; and ADFD, degradation rate of ADF.

We compared the significance of differences in the relative abundances of these overlapping taxa between the fiber degradation rate groups. Using *p* < 0.01 as the threshold, we identified overlapping taxa from the three analytical methods (Figure 6). A total of 7 taxa exhibited significant differences between the H-NDFD and L-NDFD groups (*p* < 0.01). Among these 7 taxa, 6 taxa belonged to the phylum Firmicutes (*Anaerotruncus*, *Family_XIII_UCG-002*, *Lachnoclostridium_1*, *Moryella*, *Ruminococcaceae_NK4A214_group*, and *Veillonellaceae_UCG-001*), and 1 belonged to the phylum Bacteroidetes (*Prevotellaceae_UCG-003*).

A total of 8 taxa showed significant differences between the H-ADFD and L-ADFD groups (*p* < 0.01). Of these 8 taxa, 6 belonged to the phylum Firmicutes (*Lachnospiraceae_ND3007_group*, *Family_XIII_UCG-002*, *Lachnoclostridium_1*, *Lachnospiraceae_UCG-002*, *Moryella*, and *Ruminococcaceae_NK4A214_group*), 1 to the phylum Bacteroidetes (*Prevotellaceae_UCG-003*), and 1 to the phylum Actinobacteria (*Olsenella*). These findings underscore the distinct microbial profiles associated with fiber degradation rates in the rumen of fattening sheep.

## 4 Discussion

Differences in the feed conversion efficiencies of individual sheep are complex and multifaceted and involve various functions of the digestive system. The foregut (rumen, reticulum, and omasum), true stomach, and intestines each play critical roles in the digestion, absorption, transport, and utilization of nutrients. Additionally, factors such as metabolic rate, endocrine regulation, and genetic background can influence feed conversion efficiency. However, the rumen serves as the primary fermentation chamber in ruminants, playing a key role in fiber degradation. Therefore, its direct impact on feed utilization efficiency remains a crucial area of investigation.

The significant negative correlations between ruminal NDFD and ADFD with BW and ADFI suggest that lambs with higher BW and ADFI tend to have lower fiber degradation rates. This phenomenon may be attributed to the increased energy demands associated with rapid growth and higher feed intake, which could shift the rumen microbial community and fermentation patterns toward utilizing more readily digestible energy sources rather than fibrous substrates. Previous studies have shown that lambs with higher feed intake exhibit lower fiber digestibility (Zeng et al., 2023), which is consistent with our findings. However, despite the lower fiber degradation rates in heavier and high-intake lambs, the negative correlation between higher NDFD and ADFD with FCR indicates that enhanced fiber degradation efficiency plays a crucial role in optimizing feed utilization. Lambs with higher fiber degradation rates likely extract more energy from fibrous feeds, thereby reducing the overall feed required per unit of weight gain. These findings underscore the importance of fiber degradation rate as a key factor that influences FCR and highlight its potential role in improving feed efficiency in sheep production systems.

To further analyze factors that influence ruminal fiber degradation rates, we studied the correlations between fiber degradation rates, rumen fermentation parameters, and rumen histomorphology. Although previous studies have shown that fiber degradation is related to the type of fermentation—typically, acetic fermentation occurs in high-fiber diets, whereas propionic fermentation is favored in diets rich in starch—increased dietary NDF levels lead to decreased concentrations of propionate and butyrate and increased acetate concentrations (cheng et al., 2012). However, the results of this study indicate a weak correlation between rumen fermentation parameters and fiber degradation rates. Specifically, fiber degradation rates were only significantly negatively correlated with the concentrations of valerate, with no significant differences in the concentrations of valerate between the fiber degradation rate groups. This suggests that, under the conditions of this study, the impact of rumen fermentation parameters on fiber degradation rates was limited. Generally, rumen pH is considered a key indicator of acid–base balance in the rumen and microbial activity. The normal pH range for ruminal fluid is between 5.5 and 7.5, and maintaining a stable rumen pH is crucial for the growth and reproduction of rumen microorganisms and optimal ruminal fermentation function (Eun and Beauchemin, 2005; Blanco et al., 2014). Khan et al. (2016) showed that rumen pH is associated with NDF levels; this indicates that, as dietary NDF increases, the degree of rumen fermentation decreases, resulting in a significant increase in rumen fluid pH. Yang et al. supported this conclusion, showing that diets high in NDF contain more indigestible cellulose and hemicellulose than those low in NDF, leading to an increase in rumen pH (Yang et al., 2002). Conversely, Oba and Allen (1999) showed that, when the NDF degradation rate in the rumen increases, the concentration of VFAs in the rumen also increases, leading to a decrease in pH. However, these studies primarily focused on diets with varying NDF levels rather than individual differences among animals. In our study, the pH values of sheep rumen fluid ranged from 5.29 to 6.75 and exhibited considerable individual variability, but the results indicate that pH is not the main factor that affects fiber degradation rates.

Furthermore, numerous studies have indicated that histomorphological parameters such as rumen papilla density and height can influence the absorption and transport of SCFAs (Kramer et al., 1996; Zhen et al., 2023). However, our results suggest that individual differences in rumen histomorphology are not the primary factors affecting rumen fiber degradation rates. This is largely due to the complex multidimensional factors involved in the fiber degradation process. Variations in fiber degradation efficiency are probably associated with the structure of the rumen microbial community and its metabolic functions, rather than traditional fermentation parameters and histomorphology.

Fiber degradation in the rumen primarily depends on the activity of rumen microorganisms. The interaction between rumen microorganisms and their host has become a central focus in ruminant research, as it plays a critical role in feed digestion, metabolite production, nutrient absorption, and physiological regulation of the host (Malmuthuge and Guan, 2017). Variations in the abundance, activity, and metabolic functions of specific microbial populations are closely linked to the host’s energy utilization and feed efficiency (Xue et al., 2020). Therefore, the metabolic and nutritional interplay between rumen microorganisms and the host is a key factor in improving feed conversion efficiency in ruminants. Numerous studies have investigated the relationship between rumen microbiota and feed efficiency, identifying microbial species strongly associated with feed conversion efficiency (Li et al., 2019; McLoughlin et al., 2020; Lopes et al., 2021; Na and Guan, 2022). These studies provide valuable insights into the effects of rumen microbiota on feed efficiency in ruminants.

However, feed efficiency is a complex phenotypic trait influenced by various factors, such as genetics, metabolic regulation, digestive organ structure, and microbial communities. The impact of microorganisms on feed efficiency is indirect and multi-faceted. In this study, we found a strong correlation between feed conversion efficiency and rumen fiber degradation rates, with fiber degradation dependent on the functional activity of rumen microorganisms. We investigated the microbial communities associated with fiber degradation and identified microbes indirectly related to feed efficiency. To enhance the reliability of our findings, we used a combination of correlation analysis, MWAS, and differential abundance analysis between the fiber degradation rate groups. These integrated approaches allowed us to identify microbial genera strongly linked to fiber degradation in the rumen.

The identified genera associated with NDF and ADF degradation were primarily members of the phylum Firmicutes. Firmicutes members play a pivotal role in fiber degradation, with families such as Ruminococcaceae and Lachnospiraceae being key contributors to the breakdown of cellulose and hemicellulose (White et al., 2014). Furthermore, Firmicutes members influence host physiology indirectly through the exchange of metabolites, establishing connections with other host systems to finely regulate hunger and satiety (Weimer, 2015). The results of this study underscore the significance of Firmicutes in fiber degradation.

Notably, five of the identified genera belonged to the family Lachnospiraceae, which has been shown to participate in the degradation of cellulose and hemicellulose and produce abundant SCFAs, particularly butyrate (Meehan and Beiko, 2014). Many studies have also demonstrated that the abundance of Lachnospiraceae in the rumen is positively associated with feed efficiency (Luo Guan et al., 2008; Myer et al., 2015; Li and Guan, 2017). In this study, *Moryella*, a genus within Lachnospiraceae, was significantly associated with fiber degradation rates. Other studies have also reported that *Moryella* abundance is notably higher in the rumens of low-feed-intake calves (Lee et al., 2023), and inoculating rumens with *Moryella* has been shown to potentially increase propionate production and improve feed efficiency (Hernandez-Sanabria et al., 2012). Large-scale genome-wide association studies and MWAS analyses by Wang et al. analyzed the relationships between host genetics, rumen microbiota, and phenotypes in sheep (Wang et al., 2023). Their findings indicated that mutations in host genetic loci may influence the relative abundance of *Lachnospiraceae_ND3007_group*, indirectly affecting SCFA production and BW. Our MWAS and correlation analyses were consistent with these findings and revealed a significant positive correlation between *Lachnospirace-ae_ND3007_group* and fiber degradation rates. These results suggest that Lachnospiraceae may enhance feed conversion efficiency and BW by improving fiber degradation efficiency and providing the host with essential metabolic products, offering promising applications in ruminant nutrition.

Notably, many of the genera identified in this study remain uncultured and underexplored, falling into the category of poorly understood microbial taxa. Their specific roles and metabolic mechanisms in the rumen environment remain unclear, posing challenges to conventional biochemical and metabolic studies. The rumen, as a complex and unique micro-ecosystem, hosts a diverse microbial community. Most rumen microorganisms remain uncultured, evading *in vitro* culture conditions and thus lacking detailed characterization (Gharechahi et al., 2023). The diversity and abundance of these uncultured microorganisms reflect the rumen’s ecological complexity and functional potential. These microorganisms may include core taxa essential for host–microbe metabolic interactions; moreover, they may harbor novel enzymes, metabolic pathways, and regulatory mechanisms, representing vast potential for future microbiome research and applications (Costa et al., 2020). For example, certain uncultured bacteria are capable of degrading complex polysaccharides, producing SCFAs as the host’s primary energy source. These microbial communities may outperform known functional taxa under specific environmental conditions, supporting host adaptation to particular feed types and habitats.

The challenge posed by uncultured microorganisms also represents a break-through opportunity (Tidjani Alou et al., 2020). Using advanced technologies such as metagenomics, single-cell genomics, and high-throughput culturing, researchers can elucidate the genetic makeup, metabolic potential, and interactions of these microorganisms with hosts and other microbiota (Liu et al., 2022), thereby revealing functional networks within the rumen micro-ecosystem. Such insights would not only advance our understanding of microbial contributions to fiber degradation but also lay the groundwork for developing targeted microbial products to enhance feed efficiency and reduce the environmental footprint of ruminant farming systems.

Our study highlights the significant role of ruminal fiber degradation rates in improving feed conversion efficiency and identifies specific microbial taxa associated with enhanced fiber utilization. However, the functional mechanisms underlying these microbial contributions remain to be fully explored. Future studies integrating multi-omics approaches (e.g., metagenomics, metatranscriptomics) with functional validation of key taxa could clarify the metabolic processes driving fiber degradation and feed efficiency. These investigations would ultimately inform strategies to optimize rumen function and improve the economic sustainability of sheep production.

## 5 Conclusion

This study demonstrates that ruminal fiber degradation significantly impacts feed conversion efficiency in sheep, with NDF and ADF degradation rates closely linked to feed efficiency and intake. Despite minimal contributions from ruminal tissue morphology and fermentation parameters to variations in fiber degradation rates, key microbial genera, particularly from the phylum Firmicutes and family Lachnospiraceae, were identified as critical to fiber degradation. Many of these taxa remain uncultured, highlighting opportunities for further research. Our findings offer microbial targets for improving feed efficiency and supporting more sustainable and cost-effective sheep production systems.

## Data Availability

The datasets presented in this study can be found in online repositories. The names of the repository/repositories and accession number(s) can be found in the article/Supplementary material.

## Footnotes

^1^ http://ccb.jhu.edu/software/FLASH/
