# Supplementary material for: Microbial insights into ruminal fiber degradation and feed efficiency of Hu sheep
Source: Front Microbiol. 2025 Apr 22;16:1561336. doi: 10.3389/fmicb.2025.1561336 (PMC12052710; doi:10.3389/fmicb.2025.1561336)
Supplement: Supplementary file 1 [file Table_1.docx]

Supplementary Material

Table S 1. Dietary formulation and nutrient level (air-dry basis).

| Items | Starter | Basic diet |
| --- | --- | --- |
| **Ingredients composition (% as fed)** | | |
| Bran | 6.00 | / |
| Alfalfa meal | 18.50 | / |
| Extruded corn | 22.30 | / |
| Extruded soybean | 4.00 | / |
| Corn gluten meal | 5.00 | / |
| Limestone | 0.30 | / |
| Corn | 21.00 | 32.5 |
| Premix | 1.00 | 0.50 |
| NaCl | 0.40 | 0.70 |
| Soybean meal | 21.50 | 5.00 |
| Corn germ meal | / | 18.00 |
| Corn stalks | / | 12.00 |
| Corn hulls | / | 11.20 |
| Corn cob | / | 8.00 |
| Cotton meal | / | 5.00 |
| Molasses | / | 3.30 |
| Stone powder | / | 0.80 |
| Expanded Urea | / | 0.50 |
| Bentonite | / | 1.50 |
| Baking soda | / | 1.00 |
| Total | 100.00 | 100.00 |
| **Chemical composition (%)** | | |
| Dry matter | 90.96 | 88.78 |
| Digestible energy (MJ·kg^-1^) | 13.01 | 11.11 |
| Crude protein | 19.50 | 13.09 |
| Fat | 1.33 | 1.72 |
| Neutral detergent fiber | 18.87 | 27.08 |
| Acid detergent fiber | 8.60 | 13.09 |
| Starch | 33.10 | / |
| Crude fiber | / | 9.78 |
| Nitrogen free extract | / | 55.05 |

Notes: The premix included the following per kg of the diet: 25 mg Fe as FeSO4·H2O; 40mg Zn as ZnSO_4_·H_2_O; 8mg Cu as CuSO_4_·5H_2_O; 40mg Mn as MnSO_4_·H2O; 0.3 mg I as KI; 0.2 mg Se as Na_2_SeO_3_; 0.1 mg Co as CoCl_2_; 940 IU vitamin A; 111 IU vitamin D; 20 IU vitamin E, and; 0.02 mg vitamin B_12_.
